# Oil of Eucalyptus in Scarlatina and Other Infectious Diseases

**Published:** 1890-03-29

**Authors:** 


### OIL OF EUCALYPTUS IN SCARLATINA AND OTHER INFECTIOUS DISEASES.

Some time since Dr. Murray Gibbs reported his treatment of a large number of cases of diphtheria with sprays of the oil of eucalyptus without a single death, though the mortality of those attended by other medical men during the same period was considerable. At the last meeting of the Epidemiological Society, Mr. Curgenven read a most interesting and suggestive paper on the results of his employment of this drug in a number of cases of scarlatina. He claims for it that while absolutely free from the objections inseparable from the use of poisonous disinfectants, and from those attaching to the fixed oils and fats which interfere with the functions of the skin, the oil in question, which is perfectly harmless and volatile, acts as a direct antidote to the disease, arresting its course if already developed, aborting it in the earlier stages, and protecting from infection persons in contact with the sick; indeed, as he alleges, superseding the necessity for isolation, and for subsequent disinfection of bedding, furniture, rooms, &c., by other means. We must express our opinion that taken by itself the defervescence of the fever and disappearance of the rash on the third or fifth day proves little, since recent epidemics have been on the whole of a mild type, and such early recovery is frequent enough. But that extreme swelling of the tonsils, temperatures over 104, absolute inability to swallow, and so on, should entirely subside on the second day within a few hours of the commencement of the treatment seems irresistibly to indicate a relation of the nature of cause and effect. Still more do the cases he narrated of the arrest of the disease within twenty-four hours when it had set in with high fever, sore throat, vomiting, and other symptoms presaging a severe attack, the rash either not appearing at all or being cut short when it had shown itself on a limited extent of surface. The power of resisting infection we know varies greatly in different persons, but we can scarcely thus explain the immunity enjoyed by five small children of the most susceptible age occupying the same room with one suffering from scarlatina. The cases reported by Mr. Curgenven are, perhaps, not sufficiently numerous to justify his extremely confident assertions, but they are amply enough to encourage an extensive trial of a remedy which cannot possibly do any harm. His procedure is to give a few drops of the oil internally every hour or two, to freely sprinkle the clothes, bedding, pillows, &c., to wash the entire surface of the body once or twice a day with it, and to sprinkle it lavishly about the room until the atmosphere is strongly redolent of it. He rubs it over swollen glands, and though he has not, as yet, employed it as a spray to the tonsils and throat, he admitted, in reply to our question, that it would be desirable to do so in severe cases, and certainly in all of diphtheria. He has not tried it hitherto in any other disease than scarlatina, but would advocate its employment in like manner in diphtheria, measles, small-pox, and whooping cough. The preparation that he has used is a proprietary one—Tucker's Eucalyptus Antiseptic—which contains, in addition, menthol and other volatile oils, but the pure oil of eucalyptus would probably be equally efficacious. We may here remark that though we fully recognize the antiseptic and germicidal properties of the oil, we are utterly incredulous as to the alleged value of planting the trees with a view to reducing the unhealthiness of malarial districts, in so far as it is represented to be effected by emanation from the leaves themselves. Whatever may be the effect of saturating the air of a room with the vapour of the oil, it is quite inconceivable that the atmosphere could be so impregnated by the mere presence of the trees, even if it were, which it, as a matter of fact, is not, perceptible to the smell, for the diffusion of gases alone would dilute it so far as to render it inactive. What improvement in malarious localities has sometimes followed can only be due to the enormous abstraction of water from the soil, and its dissipation by evaporation from the surface of leaves, quite independently of the presence of oil in their glands. When local conditions permit of this drying of the soil the beneficial effects are evident, but they are not observed when the water is renewed as fast as it is removed, and drainage only can achieve the desired result.

